# Effects of High-Moisture Extrusion Conditions on the Functionality and *In Vitro* Digestibility of Waxy and High-Amylose Barley Flours

**DOI:** 10.3390/foods15193452

**Published:** 2026-09-27

**Authors:** Iván Friero, Núria Elias-Santaeulalia, Francesc Puiggros, María Paz Romero, Nàdia Ortega-Olivé, Marian Moralejo

**Affiliations:** 1 AGROTECNIO-CERCA Center, Universidad de Lleida, Av. Alcalde Rovira Roure, 191, 25198 Lleida, Spain; ivan.friero@udl.cat (I.F.); nes5@alumnes.udl.cat (N.E.-S.); mariapaz.romero@udl.cat (M.P.R.); marian.moralejo@udl.cat (M.M.); 2 Unitat de Nutrició i Salut, Eurecat—Centre Tecnològic de Catalunya, 43204 Reus, Spain; francesc.puiggros@eurecat.org

**Keywords:** barley, high-moisture extrusion, resistant starch, protein digestibility, FTIR, molecular weight distribution

## Abstract

This study assessed how high-moisture extrusion (HME) alters the structural, functional, and *in vitro* digestibility properties of two barley flours with contrasting starch architectures: waxy barley (Annapurna^®^) and high-amylose barley (Hilose^®^). The flours were processed at three moisture contents (50%, 60%, and 70%) and two screw speeds (100 and 150 rpm). Moisture content emerged as the main processing factor influencing the response of both barley matrices, although the magnitude and direction of the changes depended strongly on barley composition. At 50% moisture and 150 rpm, the higher specific mechanical energy was associated with increased amylose content and resistant starch formation, with Hilose^®^ reaching 13.8% resistant starch. In contrast, processing at 70% moisture increased water absorption and rapidly digestible starch, which reached 69.6% in Annapurna^®^. Intermediate conditions (60% moisture, 100 rpm) favored β-glucan retention and were associated with higher slowly digestible starch, exceeding 40% in Hilose^®^. HME also induced matrix-dependent changes in *in vitro* protein digestibility and in starch and protein structural organization, as evidenced by FTIR analysis and protein molecular weight distribution. Overall, the results show that the functional and nutritional effects of HME depend on the interaction between processing conditions and the intrinsic starch architecture of the barley matrix, supporting the use of HME to obtain barley flours with differentiated technological and nutritional properties.

## 1. Introduction

Modern diets are frequently characterized by excessive consumption of rapidly digestible carbohydrates and proteins with limited bioavailability, contributing to the growing prevalence of obesity, type 2 diabetes, and other metabolic disorders [1]. In this context, cereal matrices represent relevant targets for process-driven modulation of macronutrient structures, such as starch, protein, and fiber. These components can be reorganized through thermo-mechanical processing, thereby modifying enzymatic accessibility and, consequently, digestibility.

Among cereals, barley (*Hordeum vulgare* L.) stands out as a promising raw material due to its high content of soluble dietary fiber, particularly β-glucans. These polysaccharides have been associated with improved glycemic control, enhanced satiety, cholesterol-lowering effects, and other metabolic benefits. Notably, barley β-glucans have been recognized by both the U.S. Food and Drug Administration and the European Food Safety Authority for their role in reducing blood cholesterol, a risk factor for coronary heart disease [2], Moreover, starch composition strongly influences digestibility and the formation of resistant starch (RS). Because RS escapes digestion in the small intestine and is fermented in the colon, it may promote beneficial gut microbiota activity and contribute to improved metabolic health [3,4].

Extrusion is a thermo-mechanical process capable of inducing controlled structural transformations in starch and proteins, thereby altering their functional and nutritional properties. Recent work on purple hull-less barley-based ready-to-eat cereals has shown that extrusion can improve nutritional and techno-functional attributes, supporting the use of barley as a functional ingredient in extruded products [5]. Nevertheless, the impact of extrusion on barley matrices depends strongly on processing conditions and grain composition, particularly starch architecture. Generally, low-moisture extrusion (LME) efficiently gelatinizes starch and denatures proteins but often involves high shear and temperature conditions that degrade β-glucans, promote protein cross-linking, and fragment starch, potentially reducing nutritional quality [6,7]. In contrast, high-moisture extrusion (HME) operates under elevated moisture conditions and generally requires lower specific mechanical energy (SME), thereby promoting starch gelatinization, protein unfolding, and matrix reorganization while potentially reducing processing severity. However, its structural and nutritional effects remain highly dependent on the food matrix. Depending on cereal grain composition and processing conditions, HME may either improve protein digestibility or promote protein aggregation and reduce enzymatic accessibility. Similarly, starch transformations during HME involve complex gelatinization and molecular rearrangements governed by moisture level, mechanical input, and intrinsic grain characteristics [8,9,10,11].

Despite the growing interest in HME on protein fractions in recent years, its application to barley flour remains insufficiently understood. In particular, limited information is available on how barley starch architecture, such as waxy versus high-amylose types, interacts with HME conditions to drive compositional changes, macromolecular reorganization, techno-functional properties, and nutrient digestibility. This knowledge gap is especially relevant for the development of barley-based ingredients with tailored hydration properties and controlled starch and protein digestibility.

The present study investigates the application of HME to two barley genotypes with contrasting starch architectures, waxy Annapurna^®^ and high-amylose Hilose^®^, with two main objectives: (i) to characterize how HME modulates the physicochemical composition and macromolecular organization of barley matrices with contrasting intrinsic structures; and (ii) to assess how the extrusion-induced compositional and structural changes influence techno-functional properties and *in vitro* starch and protein digestibility. It was hypothesized that HME promotes moisture-driven macromolecular reorganization, and that the extent and functional consequences of these structural changes are governed by the intrinsic characteristics of each barley matrix. Therefore, understanding how high-moisture extrusion modifies the structural, physicochemical, and nutritional properties of barley flours is relevant for the development of tailored barley-based ingredients with specific functional characteristics for food applications.

## 2. Materials and Methods

### 2.1. Plant Material and Chemical Composition

Barley seeds were provided by Semillas Batlle SA (Bell-Lloc d’Urgell, Lleida, Spain). Two hull-less barley varieties with high β-glucan content and contrasting starch compositions were used: Annapurna^®^, a waxy variety, and Hilose^®^, a high-amylose variety. The native Annapurna^®^ flour contained 11.4% moisture, 45.9 g/100 g dry basis (db) total starch, 15.96 g/100 g db protein, and 8.54 g/100 g db β-glucan, whereas Hilose^®^ contained 12.2% moisture, 44.01 g/100 g db total starch, 14.22 g/100 g db protein, and 9.32 g/100 g db β-glucan. Amylose accounted for 5.34% and 30.22% of total starch in Annapurna^®^ and Hilose^®^, respectively, corresponding to calculated amylopectin contents of 94.66% and 69.78% and amylose/amylopectin ratios of 0.056 and 0.433, respectively.

### 2.2. Extrusion Process

Prior to extrusion, barley grains were milled to a particle size of 1 mm using a Brabender mill (Brabender GmbH & Co. KG, Duisburg, Germany). The extrusion process was conducted using a co-rotating twin-screw extruder (TwinLab-F 20/40, C.W. Brabender^®^ GmbH & Co. KG, Duisburg, Germany) equipped with a 2 mm die. The die diameter was selected based on preliminary trials and our previous experience with the extrusion of barley-based matrices using the same extrusion system and die configuration [5,12]. This configuration provided stable material flow and consistent extrusion performance and was therefore maintained throughout all experimental runs. The screw configuration corresponded to the standard profile supplied by the manufacturer and was kept constant throughout the experimental runs. The screw profile is shown in Figure S1. The extruder comprised six heating zones with an L/D ratio of 40:1 and was equipped with a peristaltic pump to control moisture content.

Barley flours were extruded at three moisture contents (50%, 60%, and 70%) and two screw speeds (100 rpm and 150 rpm). Barrel temperatures were set at 50, 60, 70, 80, 90, and 100 °C from the feeding zone to the die, according to conditions reported to achieve effective starch gelatinization under HME [11]. A constant feed rate of 3 kg/h was applied in all experiments to ensure process stability and comparability based on preliminary trials.

Each barley variety was processed under all extrusion conditions in three independent experimental cycles. After each complete cycle, extrusion conditions were reset before initiating the next run, and the three cycles were considered independent process replicates. Die pressure (DP) and specific mechanical energy (SME) were continuously recorded during the extrusion process by the extruder software. Extrudates were dried at 40 °C for 24 h (Buffalo CS950/DB234).

### 2.3. Chemical Composition

Prior to all analytical determinations, including chemical and techno-functional analyses, both raw flour and extruded samples were ground to 0.5 mm using a Foss Cyclotec 1093™ mill (FOSS, Barcelona, Spain). Moisture content was assessed using a Moisture Tester MT-CA (Brabender GmbH & Co KG, Duisburg, Germany). Nitrogen content was quantified using the Dumas combustion method with a Nitrogen/Protein Analyzer (CN628, LECO Corporation, St. Joseph, MI, USA), following the AACC Method 46–30.01, and converted to crude protein (ISO 16634-2). The β-glucan content, total starch, and amylose content were determined using K-BGLU, K-TSTA, and K-AMYL assay kits from Megazyme (Wicklow, Ireland). All reagents used were of analytical grade. Results were expressed on a dry basis.

### 2.4. Hydration Properties

Water absorption capacity and oil absorption capacity were determined following the method described by Beuchat [13]. Briefly, one gram of flour was weighed, and 10 mL of distilled water or sunflower oil was added. Samples were vortexed and allowed to stand for 15 min before being centrifuged at 1000× *g* for 10 min (Eppendorf Centrifuge 5804 equipped with an A-4-44 rotor). The supernatant was discarded, and the wet sample in the tube was weighed.

The water absorption index (WAI), water solubility index (WSI), and swelling power (SP) were determined and calculated following the method described by Kaur & Singh [14].

### 2.5. In Vitro Starch Digestibility of Extruded Samples

The *in vitro* starch digestibility was determined according to Friero et al. [15]. The measurement of digestible starch fractions was conducted in accordance with Englyst et al. [16] into rapidly digestible starch (RDS), defined as the glucose released within 20 min, slowly digestible starch (SDS) between 20 and 120 min, and resistant starch (RS) corresponding to the non-hydrolyzed starch fraction after 120 min. To determine released reducing sugars, 0.1 mL of each sample was mixed with 0.9 mL of 96% ethanol to stop enzymatic activity and centrifuged for 10 min at 5000 *g*. The Megazyme D-Glucose Assay Kit was used to determine glucose release.

### 2.6. In Vitro Protein Digestibility

*In vitro* protein digestibility (IVPD) of extrudates and flours was carried out following the pH-drop method as described by Setia et al. [17]. In brief, a flour sample containing 62.5 mg of protein was suspended in 10 mL of distilled water and incubated at 37 °C for 1 h to allow temperature equilibration prior to enzymatic digestion. The pH of the flour suspension was adjusted to 8.0 ± 0.05 using 0.1 M NaOH or HCl. Then, an enzyme mixture consisting of 31.0 mg chymotrypsin, 16.0 mg trypsin, and 13.0 mg protease was prepared in 10.0 mL deionized water and maintained at 37 °C at pH 8.0 ± 0.05. One milliliter of the enzyme mixture was added to the flour suspension and IVPD was calculated according to the following Equation (1):

IVPD (%) = 65.66 + 18.10 × ∆pH10 min(1)

where ΔpH10 min refers to the change in pH from the initial value to the value at the end of 10 min of hydrolysis.

### 2.7. Attenuated Total Reflectance Fourier Transform Infrared Spectra Analysis (FTIR-ATR) and Molecular Weight Distribution

The FTIR-ATR spectra were recorded from 4000 to 650 cm^−1^ with a resolution of 4 cm^−1^ and 64 scans per sample in triplicate using a Jasco FT/IR-6300 spectrophotometer (Japan Spectroscopy Company, Tokyo, Japan) coupled with an ATR accessory. The spectral data were processed with JASCO Spectra Manager software v2.09.01 (JASCO Corporation, Hachioji, Tokyo, Japan). Spectra were first baseline-corrected to eliminate background contributions and were then smoothed using a Savitzky–Golay filter to reduce noise. The second derivative was used to identify peaks, and spectral deconvolution was applied to resolve overlapping bands. The absorption region between 1200 and 800 cm^−1^ was analyzed, and the peaks at 1047 cm^−1^, 1022 cm^−1^, and 995 cm^−1^ were used to estimate the short-range ordered structure of starch. Protein structural rearrangements were assessed by analyzing the amide I band (1600–1700 cm^−1^) and the amide II band (1480–1580 cm^−1^). The assignment of the main secondary structural components within the amide I region was based on the FTIR assignments reported by Kong and Yu (2007) [18]. The second-derivative spectra of the barley flour samples were examined to identify the underlying component bands and their approximate positions. Based on these spectral features, the integration limits were defined and then applied consistently to all samples. Accordingly, the following sub-ranges were evaluated: 1620–1640 cm^−1^ and 1680–1700 cm^−1^ (β-sheet), 1640–1650 cm^−1^ (random coil), 1650–1660 cm^−1^ (α-helix), and 1660–1680 cm^−1^ (β-turn).

The molecular weight distribution (MWD) of the extruded cereals was analyzed by high-performance liquid chromatography (HPLC), following the procedure described by Bougatef et al. [19]. For molecular weight calibration, the following standards were used: chymotrypsinogen (25,000 Da), ribonuclease A (13,700 Da), cytochrome c (12,380 Da), aprotinin (6511 Da), insulin (5733 Da), insulin B (3495 Da), and angiotensin (1046 Da). MWD results were expressed as relative percentages of fractions grouped according to molecular weight ranges: >13,000 Da, 13,000–10,000 Da, 10,000–3000 Da, 3000–500 Da, and <500 Da.

### 2.8. Statistical Analysis

A three-way analysis of variance (ANOVA) was performed to evaluate the effects of barley variety (V), moisture content (MC), screw speed (rpm), and their interactions (V × MC, V × rpm, MC × rpm, and V × MC × rpm) on all response variables. All factors were treated as fixed effects. Each experimental condition was independently extruded in three independent processes, which were considered biological replicates. For each biological replicate, each parameter was determined using at least two analytical replicates, with three analytical replicates being used for most parameters. Given the strong effect of barley matrix and its interactions with processing conditions observed in the three-way ANOVA, additional two-way ANOVA analyses were conducted separately for each barley variety. Within each variety, MC and screw speed were treated as fixed factors, and mean comparisons were performed using Tukey’s honestly significant difference (HSD) test. Additionally, Dunnett’s multiple-comparison test was performed to compare each extrusion condition individually with its corresponding native flour, which was considered the control. Differences were considered statistically significant at *p* < 0.05. Separate PCA analysis were then performed for each variety to examine how HME-induced compositional and structural modifications were associated with techno-functional properties and *in vitro* digestibility within each barley matrix. Correlation analysis was conducted using Pearson’s correlation coefficient. Data was analyzed using JMP software Pro 16 (SAS Institute Inc., Cary, NC, USA).

## 3. Results

### 3.1. Interaction Effects of Extrusion Parameters

The results of the three-way ANOVA are summarized in Table 1, showing the effects of variety (V), screw speed (rpm), moisture content (MC), and their interactions on the compositional, techno-functional, and digestibility parameters of the extruded products. Among the main effects, V exerted the strongest influence, highlighting the dominant role of intrinsic matrix characteristics in determining functional and digestive outcomes. Although screw speed and MC significantly affected several parameters, protein content and OAC remained unchanged.

Among the two-way interactions, V × rpm showed the least pronounced effect, suggesting that screw speed influenced both barley varieties in a similar manner, despite their contrasting matrix characteristics. This interaction was significant only for WAC and WAI and did not affect any compositional or digestibility-related parameter. In contrast, V × MC significantly affected most traits, except protein content and OAC, indicating that the effect of moisture was strongly matrix dependent. The rpm × MC interaction mainly influenced functional and digestibility-related traits, while its effect on compositional parameters was limited to the amylose content.

Given the significant effects of V and its interactions with processing variables, the dataset was further analyzed separately for each barley genotype. This approach allowed variety-specific responses to moisture content and screw speed to be evaluated for functional and digestibility-related properties.

### 3.2. Chemical Composition and Extrusion Parameters of Extruded Products and Their Corresponding Native Flours

Table 2 summarizes the extrusion parameters and physicochemical changes in the extrudates. Although both barley flours were processed under the same conditions, SME and die pressure (DP) differed between matrices, indicating differences in melt viscosity and flow behavior. The lower moisture content used (50%) generally increased SME and DP, probably due to higher melt viscosity and frictional resistance, whereas 150 rpm increased SME but reduced DP, because of higher shear input, shorter residence time, and improved material flow. Despite differences in absolute values, both processing factors induced consistent directional trends across the two barley varieties, although the magnitude of the response was matrix-dependent.

β-Glucan content was lower after extrusion in both barley varieties, with reductions of 25–29% in Annapurna^®^ and 27–36% in Hilose^®^. This agrees with Chang et al. [20], who reported that HME increased soluble β-glucans while reducing total β-glucan content. Moisture content was the main factor affecting β-glucan retention in both varieties (Tables S1 and S2), with higher values generally observed at 60–70% moisture and lower SME. This suggests a reduced structural modification, and fewer starch–β-glucan interactions through hydrogen bonding, which can otherwise form ordered structures and reduce β-glucan extractability [21].

Protein content generally decreased after extrusion compared to native flour, with a more significant effect in Annapurna^®^ compared to the Hilose^®^ variety across all extrusion conditions evaluated. However, no significant differences were observed among processing conditions within each variety (Tables S1 and S2). Thus, although SME and DP varied among treatments (Table 2), the selected thermomechanical range had a comparable effect on total protein content across treatments, suggesting relative stability of the protein fraction.

Regarding the amylose content, HME generally led to an increase within each barley variety, with the magnitude of change being matrix dependent. According to the two-way ANOVA results (Tables S1 and S2), moisture content was the main contributing factor modulating amylose content compared to screw speed. From a mechanistic standpoint, these changes are attributed to the preferential degradation of amylopectin during extrusion, whose branched α-(1→6) linkages are more susceptible to mechanical stress during extrusion, resulting in an actual increase in amylose, without indicating a rise in native amylose content [22,23]. The effect was strongest in Annapurna^®^, where the amylose content increased by more than 55%, reaching 12.25% at 50% moisture and 150 rpm (Table 2). In Hilose^®^, amylose content showed a more limited response to HME than in Annapurna^®^. Significant increases relative to the native flour were observed under several extrusion conditions, particularly at 50% and 60% MC, whereas no significant differences were detected at 70% MC (Table 2). Overall, 50% moisture combined with 150 rpm produced the greatest increase in the amylose content in both varieties, coinciding with the highest SME and DP values.

### 3.3. Hydration and Functional Properties of Extruded Products

#### 3.3.1. Water Absorption Capacity

The WAC of native flours and extrudates is presented in Table 3. Native barley flours showed distinct WAC values, reflecting differences in their intrinsic starch composition. Following HME, WAC was significantly modified in a matrix-dependent manner, with moisture content identified as the main influencing factor (Tables S1 and S2).

In Annapurna^®^, WAC increased after extrusion, with the highest values observed at 70% MC. This trend indicates that moisture content played a predominant role in determining WAC under the conditions tested. Such behavior agrees with previous studies linking starch structural modifications during extrusion with increased WAC [24,25]. Conversely, processing at 50% MC promoted partial amylopectin degradation into shorter chains, as suggested by the increase in amylose content shown in Table 2, resulting in lower WAC values in this variety. Overall, these results highlight that moisture content, rather than mechanical energy input alone, governed the extent of starch reorganization and hydration capacity in Annapurna^®^.

In contrast, Hilose^®^ showed the highest WAC values when processed at 50% MC. This amylose-rich matrix likely required higher mechanical energy to disrupt its more rigid internal organization and expose hydrophilic sites. Accordingly, the higher SME observed for Hilose^®^ at 50% MC (Table 2) enhanced starch matrix disruption and water-binding capacity, as previously described for extrusion-processed starch and high-amylose systems [26,27].

#### 3.3.2. Oil Absorption Capacity

Regarding OAC, no significant differences were detected among extrudates within either barley variety, regardless of the MC and screw speed conditions applied (Table 3, Tables S1 and S2). In the case of Annapurna^®^, WAC increased whereas OAC decreased relative to the native flour. This contrasting behavior may reflect changes in the accessibility of hydrophilic and hydrophobic sites within the extruded matrix. Starch gelatinization and reorganization of the starch–β-glucan network potentially increase the availability of hydrophilic groups for water binding while simultaneously reducing the accessibility of hydrophobic sites involved in oil binding. Thus, under the HME conditions evaluated in this study (50% and 70% MC; 100 and 150 rpm) the structural modifications induced by extrusion did not appear to result in substantial exposure of hydrophobic domains or in changes favoring oil retention, despite the differences observed in SME and DP across processing conditions.

#### 3.3.3. Water Absorption Index

Both native barley flours showed similar WAI values (Table 3). After extrusion, WAI decreased in both varieties, consistent with the reduction in β-glucan content observed after HME (Table 2). This effect can be partly associated with β-glucan degradation, as the hydrophilic structure and water-binding capacity of β-glucans make them important contributors to hydration in cereal-based matrices [28,29].

Process conditions modulated WAI in a variety-dependent manner. Two-way ANOVA results (Tables S1 and S2) showed that screw speed was the main factor influencing WAI in both varieties, whereas moisture content was significant only in Annapurna^®^. In Hilose^®^, the highest WAI values were obtained at 50–60% MC and 150 rpm, under conditions associated with relatively high SME values (Table 2). This response agrees with the rigid organization of amylose-rich starches, which require greater mechanical energy to expose hydrophilic regions involved in water binding [24]. In contrast, Annapurna^®^ reached maximum WAI at 100 rpm and 60–70% MC, probably because less severe processing conditions may preserve amylopectin structure, as supported by the lower amylose content observed under those conditions (Table 2), resulting in higher WAI in this variety.

#### 3.3.4. Water Solubility Index

Among native flours, Annapurna^®^ showed higher WSI than Hilose^®^, indicating a higher proportion of water-soluble components in the untreated matrix. After HME, WSI increased markedly in both varieties, consistent with extrusion-induced disruption of starch crystalline regions, starch degradation, and leaching of amylose and soluble polysaccharide fragments into the aqueous phase [30]. Two-way ANOVA results (Tables S1 and S2) showed that MC and the MC × screw speed interaction significantly affected WSI in Annapurna^®^, whereas both MC and screw speed were significant in Hilose^®^.

In Annapurna^®^, maximum WSI was observed at 50% MC and 150 rpm, conditions associated with higher SME values (Table 2). This promoted amylopectin fragmentation into soluble short-chain fractions, supported by the marked increase in amylose content, which contributed to the high WSI values obtained [24]. In contrast, Hilose^®^ reached maximum WSI at 60% MC and 100 rpm, suggesting greater starch solubilization under these processing conditions. At higher processing severity, amylose reassociation into more ordered or resistant structures may reduce the soluble starch fraction and limit WSI, as reported in extruded rice and buckwheat [31,32].

#### 3.3.5. Swelling Power

The SP values of native flours and extrudates are shown in Table 3. Native Annapurna^®^ showed higher SP than Hilose^®^, consistent with the greater SP of amylopectin-rich starches, whose branched structure favors granule expansion and water retention, whereas amylose limits swelling through chain interactions and more ordered structures [33,34]. After extrusion, SP responses were matrix-dependent: Hilose^®^ increased at 50% MC and 150 rpm, suggesting that higher mechanical input promoted starch disorganization and water penetration, whereas Annapurna^®^ maintained higher SP overall with no significant differences among extrusion conditions.

Collectively, hydration-related properties were mainly governed by the interaction between HME conditions and starch architecture. Hilose^®^ reached maximum WAC, WAI, WSI, and SP at 50–60% MC and 150 rpm, conditions associated with the higher SME required for modifying amylose-rich matrices. In contrast, Annapurna^®^ showed the highest hydration values at 70% MC, where water plasticization likely helped preserve the amylopectin-rich structure driving hydration functionality in this waxy barley. Thus, starch composition, particularly the waxy versus high-amylose character, largely determined the functional response of barley matrices to HME.

### 3.4. In Vitro Starch Digestibility of Extruded Products

The *in vitro* starch digestibility parameters of barley extrudates are summarized in Table 4. Extrusion distinctly altered the distribution of starch fractions, with responses depending on both matrix and processing conditions. Among the extrusion parameters, MC exerted the strongest influence (Tables S1 and S2). This finding is consistent with previous studies showing that moisture availability during HME plays a key role in modulating resistant starch (RS) formation, as it affects starch gelatinization, molecular mobility, shear transmission, and subsequent structural rearrangements [10,26]. It is important to note that RS in our extrudates likely resulted from a combination of (i) extrusion-induced gelatinization and molecular rearrangement and (ii) post-extrusion retrogradation, particularly of amylose chains during drying.

Screw speed also influenced starch fractions within each barley variety (Tables S1 and S2). Increasing rpm from 100 to 150 resulted in a general increase in RS across both barley matrices. Screw speed affects shear intensity, friction, and residence time during extrusion, and its impact on starch digestibility is matrix-dependent, varying with MC, grain composition, and screw configuration, as previously reported for extrusion-processed starch-based systems [35].

#### 3.4.1. Rapidly Digestible Starch (RDS)

The RDS generally increased at 70% MC, with waxy Annapurna^®^ barley reaching the highest value (69.6%). Higher MC promotes complete starch gelatinization under HME, disrupting crystalline structures and generating amorphous structures more accessible to enzymatic hydrolysis [35]. The lower SME observed at 70% MC in this barley (Table 2; 14.21 Wh/kg) has contributed to limiting amylopectin fragmentation, as reflected by the limited increase in the amylose content (Table 2), thereby favoring starch structures that remained highly accessible to digestive enzymes. In contrast, Hilose^®^ showed lower RDS, consistent with the higher structural rigidity of high-amylose starches, which tend to reassociate or interact with other matrix components, forming more compact structures that limit enzyme accessibility and reduce starch digestibility, and in consequence, RDS [36].

#### 3.4.2. Slowly Digestible Starch (SDS)

SDS displayed matrix-specific responses but tended to peak at 60% MC and a screw speed of 100 rpm in both barley varieties. Under these conditions, Hilose^®^ barley reached SDS values exceeding 40%, indicating that such processing conditions promoted the formation of starch structures associated with delayed enzymatic hydrolysis. In both barley varieties, the extrusion conditions favoring β-glucan retention (60% MC, 100 rpm; Table 2) coincided with the highest SDS levels, suggesting that the preservation of β-glucan integrity plays a key role in modulating starch digestibility. This relationship was also supported by the correlation matrix (Table S3), where β-glucan content showed a positive and significant association with SDS (r = 0.525, *p* = 0.017). Mechanistically, higher β-glucan content contributes to the formation of viscous or physically entangled networks with starch and proteins, limiting enzyme diffusion and slowing starch hydrolysis, thereby shifting digestion from rapid to slowly digestible fractions [37,38,39].

#### 3.4.3. Resistant Starch (RS)

RS showed the strongest dependence on processing conditions, with maximum values obtained at 50% MC and 150 rpm in both varieties. Under these conditions, RS formation was markedly increased; however, the magnitude of this response differed according to starch architecture. High-amylose Hilose^®^ barley reached the highest RS values (13.8%), whereas waxy Annapurna^®^ barley showed consistently lower RS accumulation (Table 4). Two possible explanations can account for these observations.

(i) At 50% MC, starch gelatinization may be incomplete, especially for high-amylose starches, which require higher water availability for full granule gelatinization [40]. Residual native granules could persist as RS2, increasing the total RS content [34]. However, this scenario seems unlikely, as the waxy barley variety exhibited a similar RS formation pattern under the same conditions, indicating that gelatinization was sufficiently extensive across matrices.
(ii) The combination of 50% MC and 150 rpm generated increased shear and mechanical energy, as reflected by the SME values shown in Table 2, favoring complete granule disintegration followed by molecular reorganization into retrograded starch (RS3). This interpretation aligns with Masatcioglu et al. [10], who reported lower RS at 70% moisture compared with 50–60% in high-amylose maize, highlighting the critical role of controlled shear and moisture in modulating retrogradation potential. This interpretation is also consistent with the increase in the amylose content observed under these extrusion conditions (Table 2), suggesting a shift in starch molecular organization toward a higher proportion of linear chains after HME. Such structural rearrangements are known to promote molecular reassociation and reduced enzyme accessibility, which is commonly associated with increased RS formation.

### 3.5. In Vitro Protein Digestibility in Extruded Products

The results of *in vitro* protein digestibility (IVPD) are shown in Table 4. HME significantly affected IVPD in both barley varieties, with changes depending on processing conditions and matrix composition. Although previous studies reported no significant effect of screw speed on IVPD [41,42], increasing screw speed in the present study resulted in significantly higher IVPD values in both varieties (Tables S1 and S2). Moisture content also influenced IVPD, which may be related to changes in protein conformation and intermolecular interactions during extrusion [43]. Native flours showed similar IVPD values, but extrusion induced contrasting varietal responses. In Annapurna^®^, IVPD decreased under all conditions, reaching the lowest value at 100 rpm and 70% MC. The decrease in IVPD may be associated with the formation and stabilization of protein aggregates and/or protein–starch–β-glucan interactions during HME, which could block susceptible peptide bonds and reduce their accessibility to digestive enzymes [44]. Under the high-moisture and low-screw-speed condition used, the lower mechanical energy input may have resulted in less disruption of these interactions, favoring the persistence or formation of such less accessible protein structures [45]. In contrast, Hilose^®^ generally showed similar IVPD after extrusion compared to native flour, with the highest value at 150 rpm and 60% MC. This suggests that higher mechanical energy disrupted rigid protein–starch assemblies in this high-amylose matrix, improving enzyme accessibility and enhancing protein hydrolysis during *in vitro* digestion [46]. Thus, the opposite responses of the two varieties suggest that the effect of HME on IVPD was governed not only by processing intensity but also by the intrinsic composition and structural organization of each matrix.

### 3.6. FTIR-ATR Analysis of Starch and Protein Structural Rearrangements in Extruded Products

Infrared spectroscopy in spectral regions of 1200–800 cm^−1^ and 1700–1200 cm^−1^ was used to investigate the changes in starch and protein structure, respectively (Table 5). For starch conformation, the ratios 1047/1022 cm^−1^ and 995/1022 cm^−1^ were used to estimate the degree of short-ordered structure (DO) and the degree of double helix distribution (DD) inside crystalline regions [47].

The 1047/1022 cm^−1^ ratio decreased after HME in both barley varieties, indicating a reduction in crystalline order and a shift toward more amorphous starch structures. This is typically associated with amylopectin chain shortening and reduced ability to reform ordered double helices [48]. In contrast, the 995/1022 cm^−1^ ratio decreased in Annapurna^®^ but increased in Hilose^®^, suggesting partial reorganization of linear amylose chains into double helices in the high-amylose matrix [49].

Regarding protein fraction, HME induced a decrease in α-helix content in both barley varieties at 100 rpm and 60% MC, whereas β-sheet content remained relatively stable, consistent with its structural resistance to mechanical disruption associated with strong hydrogen-bonding networks [50]. The α-helix/β-sheet ratio remained unchanged after extrusion except in Annapurna^®^, where it increased under 150 rpm and 70% MC. The observed changes in the α-helix/β-sheet ratio highlight that protein structural rearrangements during extrusion are strongly governed by the interplay between moisture, shear, and the specific grain matrix. In contrast, the random coil fraction increased in both varieties, consistent with partial protein unfolding and loss of ordered secondary structure [50,51]. Among the processing variables, MC exerted the strongest effect on random coil formation compared to screw speed, with 60% MC producing the highest increase in this fraction in both barley varieties and in wheat. The amide I/amide II ratio was the most responsive parameter to HME. Following extrusion, the amide I/amide II ratio showed matrix and process-dependent variations. It decreased significantly in Annapurna^®^ barley and showed heterogeneous responses in Hilose^®^ barley. The lowest values were observed at 150 rpm and 50% MC in both varieties. These trends reflect the balance between protein unfolding and potential reaggregation, modulated by MC, RPM, and matrix composition. Together with the MWD results, these FTIR changes suggest that protein accessibility after HME may have been influenced by matrix-specific structural rearrangements rather than by a single conformational marker.

To further elucidate whether these secondary structural modifications led to macro-molecular alterations, the molecular weight distribution (MWD) of proteins from Annapurna^®^ and Hilose^®^ varieties was evaluated by HPLC-SEC (Figure 1). Following extrusion, both varieties exhibited a noticeable redistribution of protein fractions compared to their native flours, which was highly dependent on the specific matrix and processing conditions. At 150 rpm and 50% MC, both varieties exhibited a marked reduction in the largest protein fraction (>13,000 Da), accompanied by an apparent increase in the smaller fractions (10,000–3000 Da and 3000–500 Da). This redistribution is consistent with greater protein fragmentation or depolymerization under the combination of high screw speed and relatively low moisture, where increased mechanical stress may favor the breakdown of larger protein assemblies. Additionally, the distinct response observed in the alpha-helix/beta-sheet ratio for Annapurna^®^ at 150 rpm and 70% MC correlated with a unique MWD profile. Under these conditions, Annapurna^®^ displayed a relatively greater proportion of high-molecular-weight material (>13,000 Da) than Hilose^®^, suggesting that protein aggregation or intermolecular association has been favored under the higher-moisture conditions in this matrix. Extrusion-induced protein polymerization and intermolecular association have also been reported in wheat gluten and in high-moisture plant-protein extrusion systems [46]. This interpretation is consistent with the changes observed in secondary protein structure, although the MWD analysis was descriptive and does not allow the statistical significance of these profile differences to be established. In contrast, the MWD profile of Hilose^®^ appeared comparatively stable across the molecular weight ranges examined, despite the changes observed in its FTIR-derived secondary structural parameters. This behavior may indicate a greater resistance of the protein–starch matrix of this high-amylose barley to extensive changes in protein molecular size during HME. Overall, the MWD profiles provide complementary qualitative evidence that the balance between protein fragmentation and intermolecular association depends on both barley matrix composition and extrusion conditions.

### 3.7. Principal Component Analysis of Compositional, Structural, and Functional Responses to Extrusion

Two separate PCA analyses were conducted for each barley variety to evaluate the relationships between extrusion conditions, compositional and structural changes, and their impact on functional properties and *in vitro* digestibility (Figure 2). Across both varieties, moisture content was the main driver of sample distribution, being strongly associated with PC1 (48.71% and 48.65% of the variance in Annapurna^®^ and Hilose^®^, respectively). Screw speed was mainly associated with PC2 (21.73% and 22.68%, respectively). Samples processed at 50% MC clustered consistently on the positive side of PC1, whereas higher screw speed treatments were mainly separated along PC2.

Within this multivariate space, 50% MC was associated with higher amylose content and RS formation in both varieties, consistent with greater molecular reorganization under these conditions. In contrast, samples processed at 60–70% MC were more closely associated with SDS and β-glucan content, particularly in Annapurna^®^, suggesting an association between β-glucan retention and slower starch digestion. RDS was positively associated with water absorption capacity (WAC), indicating that increased water uptake reflects a more open matrix structure that facilitates enzymatic hydrolysis.

Techno-functional properties (WAC, WAI, WSI, and SP) clustered with starch structural modifications, with Annapurna^®^ showing maximum hydration properties at 70% MC, consistent with its amylopectin-rich starch composition. In contrast, Hilose^®^ required lower moisture and higher screw speed to achieve enhanced hydration, consistent with the different structural characteristics of its high-amylose matrix.

Protein structural parameters also aligned with PCA distribution. Higher moisture levels (60–70% MC) were associated with increased random coil content, indicating partial protein unfolding. The amide I/amide II ratio showed matrix-dependent behavior, suggesting an association between protein conformational changes and IVPD that differed between the two barley matrices.

Overall, PCA confirmed that moisture content is the dominant factor governing structural and functional variability in extruded barley, followed by screw speed, and that responses are strongly dependent on starch architecture and protein matrix organization. This multivariate analysis reinforces the process–structure–function relationships underlying HME-induced modifications in cereal systems.

## 4. Conclusions

This study demonstrated that HME can modify the structural, physicochemical, and nutritional properties of barley flour in a matrix-dependent manner, with moisture content emerging as the most influential processing variable. The two barley matrices exhibited similar directional responses to MC and screw speed in terms of SME and DP, whereas their compositional differences resulted in distinct responses at the starch and protein levels. Conditions combining 50% MC with a screw speed of 150 rpm generated the highest SME and were associated with increases in amylose content and greater RS formation. In contrast, 70% MC promoted greater starch gelatinization and enhanced water absorption properties and RDS, particularly in waxy Annapurna^®^. Intermediate moisture conditions (60% MC) combined with 100 rpm favored β-glucan retention, which was associated with higher SDS, highlighting the potential role of β-glucan in limiting starch hydrolysis. HME also induced matrix-specific changes in protein structure, as evidenced by FTIR-derived alterations in secondary structure and the redistribution of protein molecular weight fractions. These changes suggest that the effect of extrusion on IVPD depended on the balance between protein unfolding, fragmentation, aggregation, and interactions within the starch–β-glucan matrix, with Annapurna^®^ showing a reduction in IVPD across all extrusion conditions relative to its native flour. At the starch structural level, changes in the 1047/1022 cm^−1^ and 995/1022 cm^−1^ ratios further indicated modifications in short-range order and starch organization associated with extrusion and RS formation. PCA reinforced the matrix-dependent nature of these responses and highlighted the close relationship between processing conditions, molecular structure, and resulting functionality.

Overall, these findings demonstrate that HME can serve as a versatile tool for tailoring barley flour properties by combining the intrinsic characteristics of the starting matrix with controlled processing conditions. Rather than generating a single type of modified flour, the use of barley matrices with different starch compositions, such as waxy and high-amylose varieties, enables the production of ingredients with differentiated structural, technological, and nutritional characteristics. Such tailored flours offer practical applicability in the development of barley-based foods with specific functional or nutritional profiles. These applications highlight the potential of HME as a structuring technology for designing barley ingredients aligned with targeted food functionalities and health-oriented product development.

## Figures and Tables

**Figure 1:**
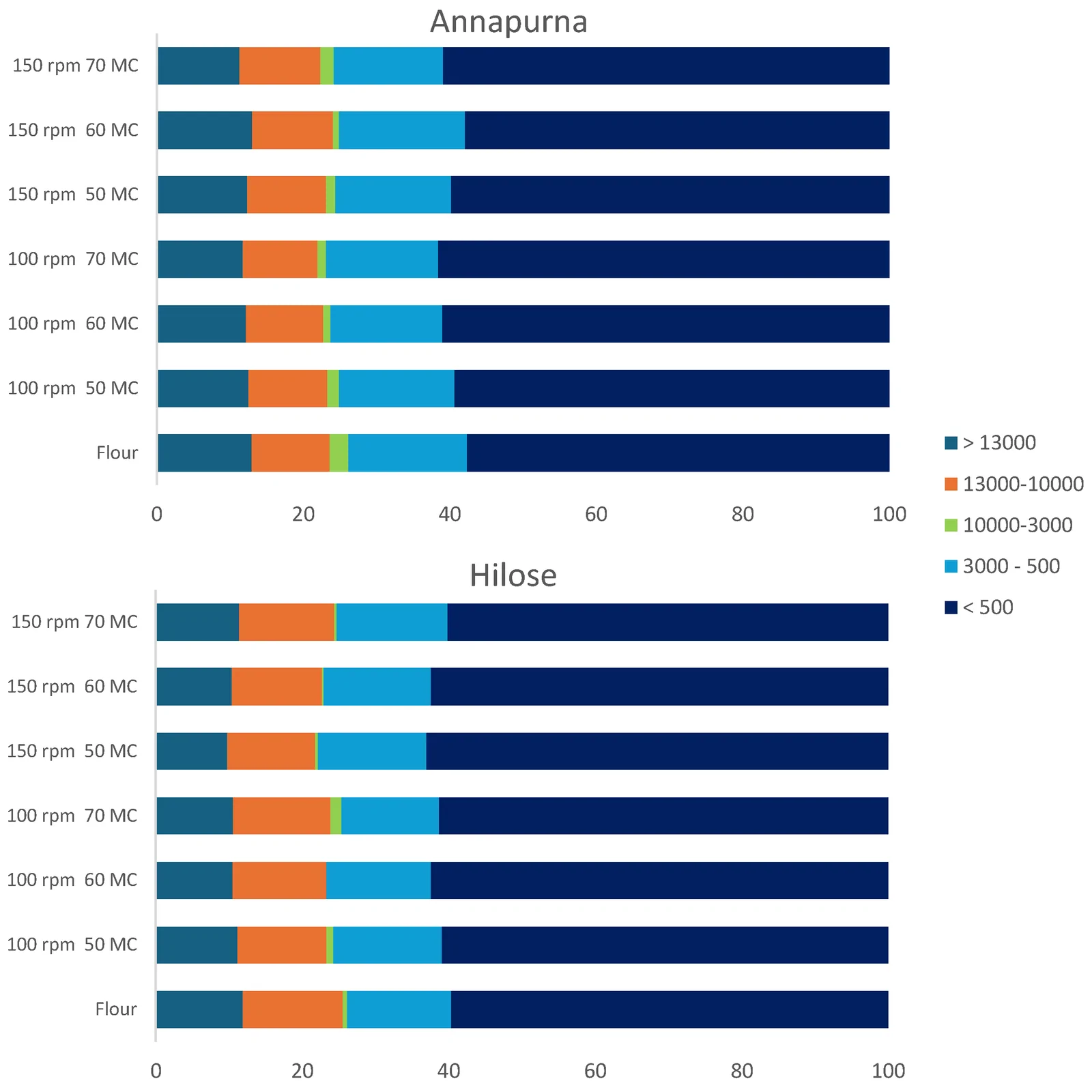
Molecular weight distribution (MWD) of proteins in native flour and extruded products processed under different conditions. Results are expressed as percentage of total protein detected by gel permeation chromatography. Protein fractions were categorized according to molecular weight as follows: >13,000 Da (blue), 13,000–10,000 Da (orange), 10,000–3000 Da (green), 3000–500 Da (light blue), and <500 Da (dark blue).

**Figure 2:**
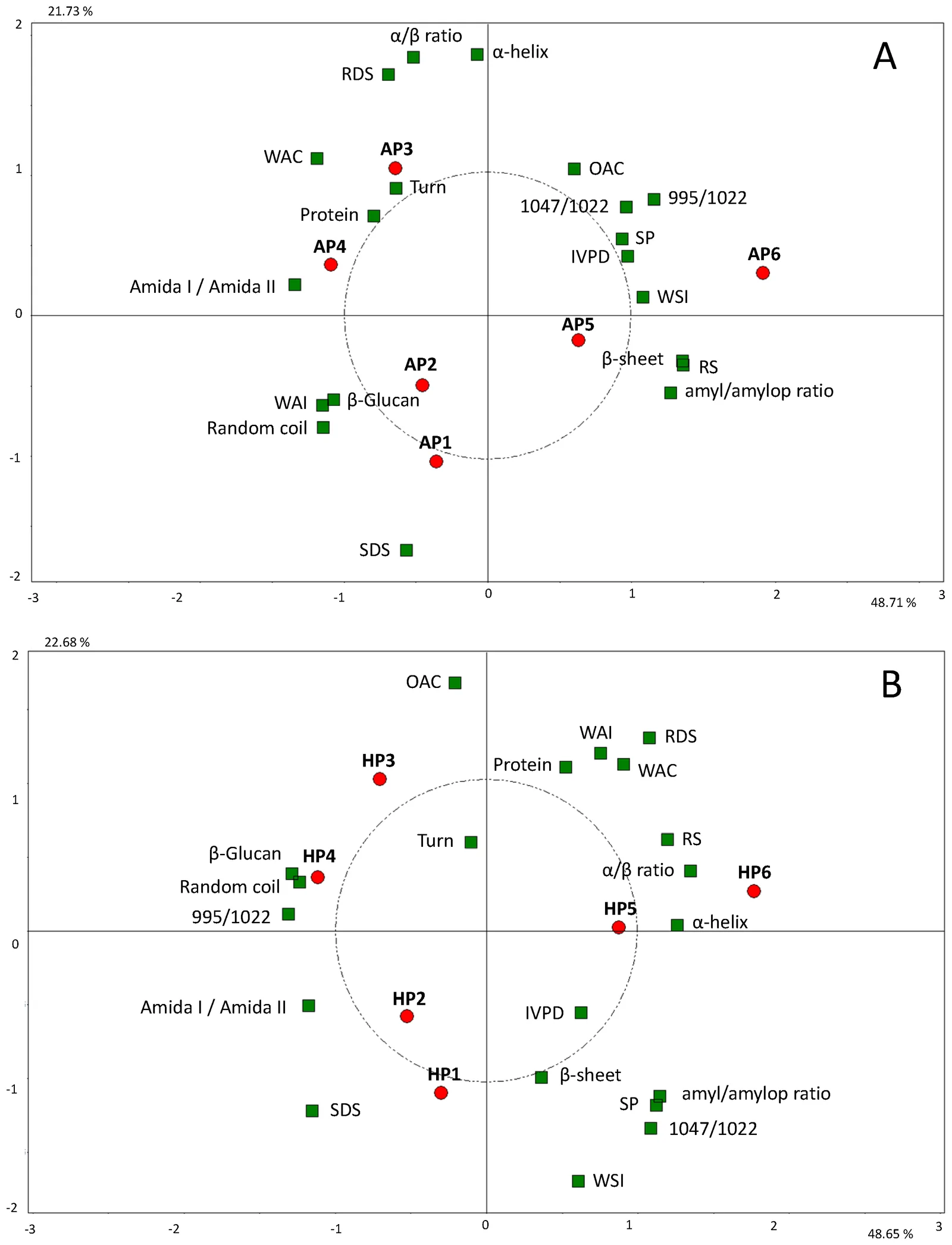
Principal component analysis of extrudates for each grain. (**A**): Annapurna^®^ (A) and (**B**): Hilose^®^ (H). Extrusion conditions: P1: 100 rpm, 60% moisture; P2: 150 rpm, 60% moisture; P3: 150 rpm, 70% moisture; P4: 100 rpm, 70% moisture; P5: 100 rpm, 50% moisture; P6: 150 rpm, 50% moisture. amyl: amylose; amylop: amylopectin; WAC: water absorption capacity; OAC: oil absorption capacity; WAI: water absorption index; WSI: water solubility index; SP: swelling power; RDS: rapid digestible starch; SDS: slow digestible starch; RS: resistant starch; IVPD: *in vitro* protein digestibility.

**Table 1:** Analysis of variance (ANOVA) showing the effects of variety, moisture content, screw speed, and their interactions on the studied parameters of the extruded products.

Source	DF	β-Glucan (g/100 g)		Protein (g/100 g)		Amylose Content (%)		WAC (g/g)		OAC (g/g)		WAI (g/g)		WSI (g/100 g)		SP (g/g)		RDS (%)		SDS (%)		RS (%)		IVPD (%)	
		SS	*p*	SS	*p*	SS	*p*	SS	*p*	SS	*p*	SS	*p*	SS	*p*	SS	*p*	SS	*p*	SS	*p*	SS	*p*	SS	*p*
V	1	0.21	0.003	12.93	<0.0001	2812	<0.0001	32.50	<0.0001	0.085	<0.0001	5.24	<0.0001	1070	<0.0001	50.72	<0.0001	651.2	<0.0001	108.4	<0.0001	228.3	<0.0001	9.63	<0.0001
RPM	1	0.09	0.034	0.89	0.071	3.97	0.0002	0.015	0.162	0.001	0.533	0.006	0.501	8.61	0.008	0.002	0.909	58.2	0.002	115.1	<0.0001	9.61	<0.0001	23.07	<0.0001
MC	2	0.81	<0.0001	0.09	0.817	32.19	<0.0001	0.57	<0.0001	0.004	0.099	0.075	0.079	50.47	<0.0001	0.535	0.187	161.6	<0.0001	294.9	<0.0001	76.92	<0.0001	10.36	<0.0001
V × RPM	1	0.06	0.082	0.13	0.470	0.32	0.156	0.04	0.031	0.001	0.834	0.496	<0.0001	0.39	0.510	0.004	0.865	1.2	0.462	0.02	0.915	0.88	0.053	0.005	0.859
V × MC	2	0.16	0.025	0.49	0.367	0.89	0.078	0.91	<0.0001	0.002	0.869	0.295	0.012	35.85	<0.0001	0.393	0.275	199.6	<0.0001	125.2	<0.0001	19.88	<0.0001	1.99	0.016
RPM × MC	2	0.08	0.126	1.12	0.126	2.42	0.005	0.07	0.023	0.001	0.475	0.019	0.467	47.75	<0.0001	0.284	0.384	12.2	0.091	15.6	0.043	4.88	0.001	0.21	0.553
V × RPM × MC	2	0.18	0.016	0.26	0.577	0.09	0.720	0.06	0.033	0.006	0.052	0.001	0.991	9.36	0.021	0.068	0.786	27.6	0.011	26.3	0.009	1.59	0.043	1.32	0.048
Error	18																								

ANOVA table showing the degrees of freedom (DF), sum of squares (SS), and corresponding significance level (*p*-value) for the effects of variety (V), screw speed (RPM), moisture content (MC), and their interactions on the studied parameters. WAC: water absorption capacity; OAC: oil absorption capacity; WAI; water absorption index; WSI: water solubility index; SP: swelling power; RDS: rapid digestible starch; SDS: slow digestible starch; RS: resistant starch; IVPD: *in vitro* protein digestibility.

**Table 2:** Extrusion parameters and physicochemical composition of extruded products and their corresponding native flours.

Variety	RPM	MC	SME (Wh/kg)				DP (Bar)				β-Glucan (g/100 g db)				Protein (g/100 g db)				Amylose Content (%)			
Annapurna^®^	Flour										8.54	±	0.23	A	15.96	±	0.10	A	5.34	±	0.36	B
	100	50	31.00	±	2.97	b	2.81	±	0.11	a	6.04	±	0.11	b ***	14.04	±	0.00	a **	10.49	±	0.39	b ***
	100	60	16.00	±	1.87	d	1.88	±	0.08	c	6.45	±	0.11	a ***	13.79	±	0.45	a ***	10.47	±	0.45	b ***
	100	70	14.21	±	1.81	d	1.71	±	0.04	d	6.20	±	0.02	ab ***	14.03	±	0.63	a **	8.30	±	0.23	c ***
	150	50	60.00	±	3.74	a	2.69	±	0.09	b	5.99	±	0.14	b ***	13.73	±	0.10	a ***	12.25	±	0.49	a ***
	150	60	25.50	±	1.29	c	1.91	±	0.05	c	6.29	±	0.24	ab ***	14.43	±	0.28	a **	10.56	±	0.14	b ***
	150	70	24.64	±	1.08	c	1.64	±	0.08	d	6.34	±	0.07	ab ***	14.41	±	0.00	a **	9.59	±	0.09	bc ***
Hilose^®^	Flour										9.32	±	0.20	A	14.22	±	0.09	B	30.22	±	0.46	A
	100	50	25.20	±	2.82	b	9.60	±	0.60	a	6.37	±	0.21	a ***	12.88	±	0.91	a	32.44	±	0.24	a **
	100	60	24.22	±	1.09	bc	5.08	±	0.48	c	6.33	±	0.13	ab ***	12.16	±	0.07	a *	32.63	±	0.27	a **
	100	70	22.73	±	2.15	c	4.99	±	0.38	c	6.77	±	0.01	a ***	11.98	±	0.94	a *	29.83	±	0.34	b
	150	50	40.38	±	3.07	a	8.32	±	0.47	b	5.90	±	0.13	b ***	12.77	±	0.25	a	33.77	±	0.59	a ***
	150	60	38.90	±	0.99	a	5.43	±	0.26	c	6.43	±	0.01	a ***	12.64	±	0.35	a	32.54	±	0.47	a **
	150	70	39.50	±	1.18	a	5.03	±	0.59	c	6.53	±	0.13	a ***	13.20	±	0.37	a	30.34	±	0.42	b

Values are presented as mean ± standard deviation. Statistical differences were also determined using Tukey–Kramer’s HSD test (α = 0.05). Uppercase letters denote significant differences among raw flours, whereas lowercase letters denote significant differences among extrusion conditions within each grain. Asterisks indicate significant differences from the native flour according to Dunnett’s multiple-comparison test: * = *p* < 0.05, ** = *p* < 0.01, and *** = *p* < 0.001. SS: screw speed, MC: moisture content, SME: specific mechanical energy, DP: die pressure. db: dry basis.

**Table 3:** Functional properties of extruded products and their native flours.

Variety	RPM	MC	WAC (g/g)				OAC (g/g)				WAI (g/g)				WSI (g/100 g)				SP (g/g)			
Annapurna^®^	Flour		2.72	±	0.06	B	1.59	±	0.13	A	9.86	±	0.04	A	8.56	±	0.44	A	10.78	±	0.09	A
	100	50	4.05	±	0.09	c ***	0.78	±	0.05	a ***	7.37	±	0.11	ab ***	29.06	±	0.49	ab ***	10.39	±	0.24	a
	100	60	4.15	±	0.21	bc ***	0.80	±	0.01	a ***	7.76	±	0.06	a ***	29.27	±	1.56	ab ***	10.46	±	0.40	a
	100	70	4.69	±	0.05	a ***	0.80	±	0.04	a ***	7.76	±	0.18	a ***	26.93	±	0.28	bc ***	10.32	±	0.14	a
	150	50	3.90	±	0.02	c ***	0.83	±	0.03	a ***	7.12	±	0.04	b ***	32.60	±	1.99	a ***	10.57	±	0.36	a
	150	60	4.49	±	0.08	ab ***	0.75	±	0.01	a ***	7.38	±	0.05	ab ***	23.03	±	0.04	c ***	10.26	±	0.79	a
	150	70	4.89	±	0.08	a ***	0.81	±	0.04	a ***	7.44	±	0.10	ab ***	26.81	±	1.15	bc ***	10.47	±	0.41	a
Hilose^®^	Flour		3.01	±	0.22	A	1.44	±	0.14	A	9.67	±	0.05	A	6.67	±	0.31	B	5.84	±	0.10	B
	100	50	2.18	±	0.02	a ***	0.69	±	0.00	a ***	6.46	±	0.11	a ***	15.50	±	0.71	b ***	7.65	±	0.20	ab ***
	100	60	1.90	±	0.04	c ***	0.65	±	0.02	a ***	6.36	±	0.01	a ***	18.25	±	0.39	a ***	7.83	±	0.09	a ***
	100	70	2.07	±	0.00	ab ***	0.67	±	0.01	a ***	6.40	±	0.03	a ***	12.20	±	0.78	c ***	7.29	±	0.10	b ***
	150	50	2.15	±	0.01	ab ***	0.66	±	0.04	a ***	6.78	±	0.17	a ***	15.19	±	0.27	b ***	8.04	±	0.23	a ***
	150	60	1.90	±	0.08	c ***	0.67	±	0.01	a ***	6.54	±	0.05	a ***	14.18	±	0.95	bc ***	7.57	±	0.08	ab ***
	150	70	2.00	±	0.00	bc ***	0.71	±	0.03	a ***	6.68	±	0.19	a ***	12.22	±	0.30	c ***	7.14	±	0.19	b ***

Values are presented as mean ± standard deviation. Statistical differences were also determined using Tukey–Kramer’s HSD test (α = 0.05). Uppercase letters denote significant differences among raw flours, whereas lowercase letters denote significant differences among extrusion conditions within each grain. Asterisks indicate significant differences from the native flour according to Dunnett’s multiple-comparison test: * = *p* < 0.05, ** = *p* < 0.01, and *** = *p* < 0.001. RPM: revolutions per minute, MC: moisture content, WAC: water absorption capacity, OAC: oil absorption capacity, WAI: water absorption index, WSI: water solubility index, SP: swelling power.

**Table 4:** Digestible parameters of extruded products and their native flours.

Variety	RPM	MC	RDS (%)				SDS (%)				RS (%)				IVPD (%)			
Annapurna^®^	Flour		16.87	±	1.06	A	49.25	±	1.78	A	33.87	±	3.32	B	76.88	±	0.77	A
	100	50	60.85	±	0.92	c ***	32.12	±	1.13	ab ***	7.04	±	0.21	b ***	73.90	±	0.13	bc **
	100	60	61.45	±	0.90	c ***	35.09	±	0.30	a ***	3.46	±	0.60	d ***	72.99	±	0.13	cd ***
	100	70	68.21	±	1.34	ab ***	30.64	±	1.76	abc ***	1.15	±	0.42	e ***	72.09	±	0.64	d ***
	150	50	64.18	±	0.85	bc ***	26.65	±	1.05	c ***	9.16	±	0.19	a ***	75.52	±	0.38	a
	150	60	64.73	±	0.51	bc ***	30.12	±	0.92	abc ***	5.15	±	0.40	c ***	74.80	±	0.13	ab **
	150	70	69.61	±	1.55	a ***	28.11	±	1.86	bc ***	2.28	±	0.31	de ***	74.44	±	0.38	ab **
Hilose^®^	Flour		15.63	±	0.55	A	36.70	±	0.71	B	47.72	±	4.11	A	75.71	±	0.38	A
	100	50	58.29	±	2.33	a ***	30.71	±	1.60	b ***	11.00	±	0.73	b ***	74.44	±	0.13	b
	100	60	49.30	±	2.12	b ***	41.03	±	1.85	a **	9.67	±	0.27	b ***	74.71	±	0.51	b
	100	70	50.34	±	0.92	b ***	39.03	±	0.32	a *	10.63	±	0.60	b ***	73.53	±	0.38	b **
	150	50	62.21	±	2.20	a ***	23.99	±	2.03	c ***	13.80	±	0.17	a ***	76.79	±	0.38	a
	150	60	48.37	±	0.32	b ***	41.55	±	0.32	a **	10.08	±	0.64	b ***	77.24	±	0.51	a *
	150	70	58.02	±	1.46	a ***	31.91	±	1.54	b ***	10.07	±	0.09	b ***	74.62	±	0.64	b

Values are presented as mean ± standard deviation. Statistical differences were also determined using Tukey–Kramer’s HSD test (α = 0.05). Uppercase letters denote significant differences among raw flours, whereas lowercase letters denote significant differences among extrusion conditions within each grain. Asterisks indicate significant differences from the native flour according to Dunnett’s multiple-comparison test: * = *p* < 0.05, ** = *p* < 0.01, and *** = *p* < 0.001. RPM: revolutions per minute, MC: moisture content, RDS: rapid digestible starch, SDS: slow digestible starch, RS: resistant starch, IVPD: *In vitro* protein digestibility.

**Table 5:** FTIR analysis of starch conformation and protein secondary structure in native flours and extruded products.

Variety	RPM	MC	1047/1022 cm^−1^				995/1022 cm^−1^				α-Helix (%)				β-Sheet (%)				α/β Ratio				Random Coil (%)				Turn (%)				Amide I/Amide II			
Annapurna^®^	Flour		0.78	±	0.01	A	1.00	±	0.01	A	12.92	±	0.03	A	55.16	±	0.07	A	0.23	±	0.00	A	19.13	±	0.16	A	12.80	±	0.14	A	3.18	±	0.11	A
	100	50	0.72	±	0.00	a ***	0.98	±	0.00	a ***	12.13	±	0.11	a *	57.16	±	0.22	a ***	0.21	±	0.00	ab	18.50	±	0.21	b	12.20	±	0.05	b ***	2.98	±	0.03	a **
	100	60	0.73	±	0.01	a ***	0.97	±	0.00	a ***	11.14	±	0.37	b ***	56.03	±	0.82	a	0.20	±	0.01	c	20.38	±	0.86	a	12.37	±	0.09	ab **	3.00	±	0.02	a **
	100	70	0.73	±	0.00	a ***	0.97	±	0.00	a ***	12.21	±	0.09	a	55.57	±	0.19	a	0.22	±	0.00	a	19.92	±	0.15	ab	12.29	±	0.05	ab ***	3.05	±	0.07	a
	150	50	0.74	±	0.02	a *	0.98	±	0.00	a ***	11.82	±	0.14	ab ***	57.35	±	0.4	a ***	0.21	±	0.00	bc	18.59	±	0.52	b	12.24	±	0.12	ab ***	2.85	±	0.08	a ***
	150	60	0.72	±	0.03	a ***	0.97	±	0.01	a ***	11.50	±	0.73	ab ***	56.10	±	0.85	a	0.20	±	0.01	bc	20.19	±	1.86	ab	12.21	±	0.30	b ***	2.97	±	0.08	a **
	150	70	0.73	±	0.01	a ***	0.97	±	0.00	a ***	12.28	±	0.05	a	55.63	±	1	a	0.22	±	0.00	a	19.60	±	1.01	ab	12.49	±	0.05	a	3.03	±	0.03	a
Hilose^®^	Flour		0.75	±	0.00	B	0.97	±	0.01	A	11.84	±	0.39	B	55.29	±	0.59	A	0.21	±	0.01	B	20.34	±	0.72	A	12.54	±	0.41	A	3.08	±	0.14	A
	100	50	0.74	±	0.01	a	0.97	±	0.01	b	12.86	±	0.74	a	57.05	±	1.5	a	0.23	±	0.01	a	19.97	±	1.54	b	10.12	±	0.57	b **	2.96	±	0.16	ab
	100	60	0.74	±	0.00	a	0.98	±	0.00	ab	11.46	±	0.37	b	55.78	±	0.19	a	0.21	±	0.01	b	20.65	±	0.67	ab	12.11	±	1.09	a	3.09	±	0.18	a
	100	70	0.73	±	0.01	a *	0.98	±	0.01	a	11.17	±	0.15	b	54.41	±	0.34	a	0.21	±	0.00	b	21.88	±	0.04	ab	12.53	±	0.14	a	2.98	±	0.11	ab
	150	50	0.74	±	0.01	a	0.97	±	0.00	b	12.42	±	0.03	ab	54.82	±	0.47	a	0.23	±	0.00	a	20.17	±	0.34	ab	12.58	±	0.10	a	2.72	±	0.08	b *
	150	60	0.74	±	0.01	a	0.99	±	0.00	a	11.61	±	0.66	ab	56.07	±	0.55	a	0.21	±	0.01	b	21.94	±	0.93	a	10.38	±	1.12	b *	3.10	±	0.12	a
	150	70	0.73	±	0.00	a *	0.99	±	0.01	a	11.40	±	0.38	b	54.97	±	1.24	a	0.21	±	0.01	b	21.58	±	1.62	ab	12.05	±	0.96	a	3.10	±	0.10	a

Values are presented as mean ± standard deviation. Statistical differences were also determined using Tukey–Kramer’s HSD test (α = 0.05). Uppercase letters denote significant differences among raw flours, whereas lowercase letters denote significant differences among extrusion conditions within each grain. Asterisks indicate significant differences from the native flour according to Dunnett’s multiple-comparison test: * = *p* < 0.05, ** = *p* < 0.01, and *** = *p* < 0.001. RPM: revolutions per minute, MC: moisture content.

## Data Availability

The original contributions presented in this study are included in the article/Supplementary Materials. Further inquiries can be directed to the corresponding author.

## Supplementary Materials

The following supporting information can be downloaded at https://www.mdpi.com/article/10.3390/foods15193452/s1. Figure S1: Screw profile used for the high-moisture extrusion. Table S1. Analysis of variance (ANOVA) of Annapurna® variety showing the effects of moisture content, screw speed, and their interaction on the studied parameters. Table S2. Analysis of variance (ANOVA) of Hilose® variety showing the effects of moisture content, screw speed, and their interaction on the studied parameters. Table S3. Correlation matrix among physicochemical, functional, digestive, and structural parameters of extruded products.
