# Supplementary material for: Effects of High-Moisture Extrusion Conditions on the Functionality and In Vitro Digestibility of Waxy and High-Amylose Barley Flours
Source: Foods. 2026 Sep 27;15(19):3452. doi: 10.3390/foods15193452 (PMC13664589; doi:10.3390/foods15193452)
Supplement: Supplementary file 1 [file foods-15-03452-s001.zip › foods-4518734-supplementary.pdf]

Effects of High-Moisture Extrusion Conditions on the Functionality and *In vitro* Digestibility of Waxy and High-Amylose Barley Flours

Iván Friero [1], Núria Elias-Santaeulalia [1], Francesc Puiggros [2], María Paz Romero [1], Nàdia Ortega-Olivé [1,*] and Marian Moralejo [1]

[1] AGROTECNIO-CERCA Center, Universidad de Lleida, Av. Alcalde Rovira Roure, 191, 25198 Lleida, Spain; ivan.friero@udl.cat (I.F.); nes5@alumnes.udl.cat (N.E.-S.); mariapaz.romero@udl.cat (M.P.R.); marian.moralejo@udl.cat (M.M.)

[2] Unitat de Nutrició i Salut, Eurecat—Centre Tecnològic de Catalunya, 43204 Reus, Spain; francesc.puiggros@eurecat.org

* Correspondence: nadia.ortega@udl.cat

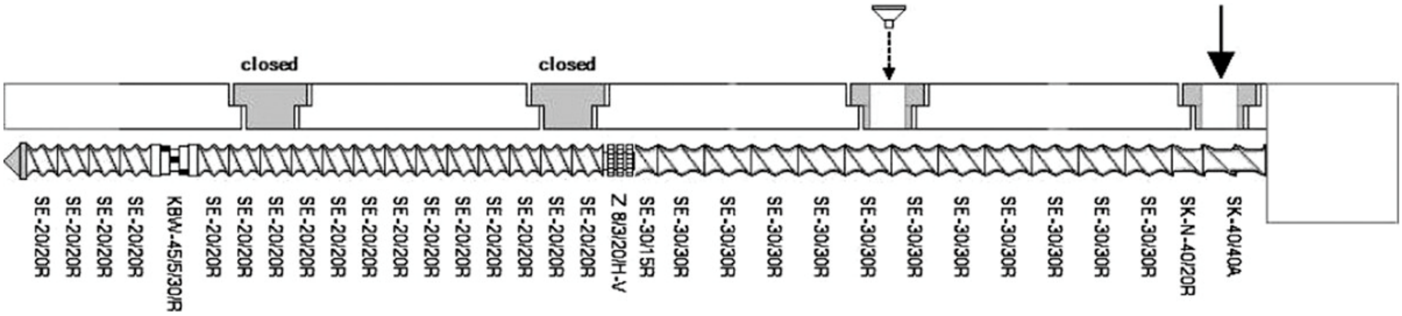

Figure S1: Screw profile used for the high-moisture extrusion.

Table S1. Analysis of variance (ANOVA) of **Annapurna** variety showing the effects of moisture content, screw speed, and their interaction on the studied parameters.

Source	DF	β-glucan (g/100g)		Protein (g/100g)		Amylose content (%)		WAC (g/g)		OAC (g/g)		WAI (g/g)		WSI (g/100g)		SP (g/g)		RDS (%)		SDS (%)		RS (%)		IVPD (%)	
		SS	*p*	SS	*p*	SS	*p*	SS	*p*	SS	*p*	SS	*p*	SS	*p*	SS	*p*	SS	*p*	SS	*p*	SS	*p*	SS	*p*
RPM	1	0.002	0.747	0.173	0.264	3.27	**0.002**	0.051	0.082	0.001	0.802	0.304	**0.002**	2.65	0.209	0.006	0.884	21.37	**0.005**	55.94	**0.001**	8.16	**0.003**	11.18	**<.0001**
MC	2	0.395	**0.003**	0.229	0.419	12.16	**0.0001**	1.347	**0.0001**	0.002	0.392	0.310	**0.004**	50.84	**<.0001**	0.031	0.943	100.10	**0.0003**	27.76	**0.018**	82.41	**<.0001**	4.22	**0.004**
RPM x MC	2	0.061	0.236	0.488	0.199	1.49	**0.031**	0.126	**0.045**	0.005	0.182	0.009	0.676	48.79	**<.0001**	0.097	0.831	2.42	0.404	4.92	0.298	0.49	0.261	0.28	0.383

ANOVA table showing the degree of freedom (DF), sum of squares (SS) and corresponding significance level (p-value) for the effects of screw speed (RPM), moisture content (MC), and their interaction on the studied parameters. WAC: water absorption capacity, OAC: Oil absorption capacity, WAI; Water absorption index, WSI: Water solubility index, SP: Swelling power, RDS: Rapid digestible starch, SDS: Slow digestible starch, RS: Resistant starch, IVPD: *in vitro* protein digestibility.

Table S2. Analysis of variance (ANOVA) of **Hilose** variety showing the effects of moisture content, screw speed, and their interaction on the studied parameters.

Source	DF	β-glucan (g/100g)		Protein (g/100g)		Amylose content (%)		WAC (g/g)		OAC (g/g)		WAI (g/g)		WSI (g/100g)		SP (g/g)		RDS (%)		SDS (%)		RS (%)		IVPD (%)	
		SS	*p*	SS	*p*	SS	*p*	SS	*p*	SS	*p*	SS	*p*	SS	*p*	SS	*p*	SS	*p*	SS	*p*	SS	*p*	SS	*p*
RPM	1	0.131	**0.023**	0.843	0.166	1.02	**0.047**	0.003	0.205	0.001	0.488	0.198	**0.009**	6.35	**0.007**	0.001	0.924	37.98	**0.012**	59.15	**0.002**	8.16	**0.003**	11.89	**<.0001**
MC	2	0.575	**0.002**	0.358	0.615	20.92	**<.0001**	0.138	**0.0002**	0.002	0.187	0.059	0.193	35.48	**<.0001**	0.888	**0.001**	261.10	**0.0003**	392.34	**<.0001**	82.41	**<.0001**	8.14	**0.002**
RPM x MC	2	0.182	**0.033**	0.896	0.335	1.02	0.119	0.003	0.441	0.003	0.175	0.011	0.684	10.31	**0.006**	0.255	**0.035**	37.34	**0.034**	37.01	**0.017**	0.49	0.261	1.24	0.125

ANOVA table showing the degree of freedom (DF), sum of squares (SS) and corresponding significance level (p-value) for the effects of screw speed (RPM), moisture content (MC), and their interaction on the studied parameters. WAC: water absorption capacity, OAC: Oil absorption capacity, WAI; Water absorption index, WSI: Water solubility index, SP: Swelling power, RDS: Rapid digestible starch, SDS: Slow digestible starch, RS: Resistant starch, IVPD: *in vitro* protein digestibility.

Table S3. Correlation matrix among physicochemical, functional, digestive, and structural parameters of extruded products.

	β-glucan	Amylose content	Protein	WAC	OAC	WAI	WSI	SP	IVPD	RDS	SDS	RS	1047/ 1022	995/ 1022	α-helix	β-sheet	Random coil	Turn	α/β ratio	Amide I/ Amide II
β-glucan	1,0000	0,1898	-0,0900	-0,2029	-0,2057	-0,2877	-0,3970	-0,3686	-0,2417	-0,3621	0,5251	-0,1312	-0,0264	0,1107	-0,2591	-0,2437	0,4786	-0,3248	-0,1741	0,4841
Amylose content	0,4228	1,0000	-0,8270	-0,9800	-0,8898	-0,9212	-0,8998	-0,9541	0,5175	-0,7525	0,3561	0,8564	0,3789	0,4046	0,0705	-0,1785	0,4491	-0,4722	0,1367	0,1217
Protein	0,7060	<,0001	1,0000	0,8505	0,7797	0,7722	0,6886	0,8123	-0,3098	0,8065	-0,5240	-0,7137	-0,4132	-0,3330	0,1308	0,1242	-0,3479	0,2504	0,0904	-0,1928
WAC	0,3910	<,0001	<,0001	1,0000	0,8615	0,9225	0,8638	0,9466	-0,4773	0,8237	-0,4356	-0,8717	-0,3471	-0,4769	0,0428	0,1246	-0,4483	0,4586	-0,0000	-0,1581
OAC	0,3844	<,0001	<,0001	<,0001	1,0000	0,7902	0,8532	0,8269	-0,4334	0,7469	-0,4424	-0,7224	-0,4191	-0,2661	-0,0540	0,2427	-0,4657	0,4017	-0,1448	-0,1196
WAI	0,2187	<,0001	<,0001	<,0001	<,0001	1,0000	0,8123	0,8881	-0,4790	0,7795	-0,4008	-0,8412	-0,3226	-0,5225	-0,0996	0,0818	-0,3668	0,4974	-0,1278	-0,2089
WSI	0,0830	<,0001	0,0002	<,0001	<,0001	<,0001	1,0000	0,9315	-0,3591	0,6607	-0,3671	-0,6738	-0,2054	-0,3870	-0,0373	0,3479	-0,5733	0,4104	-0,1679	-0,1926
SP	0,1001	<,0001	<,0001	<,0001	<,0001	<,0001	<,0001	1,0000	-0,3649	0,7541	-0,4254	-0,7599	-0,3101	-0,4687	-0,0071	0,2104	-0,4501	0,4110	-0,0805	-0,2358
IVPD	0,3046	0,0096	0,1406	0,0184	0,0344	0,0179	0,0848	0,0795	1,0000	-0,3150	-0,0516	0,6461	0,4725	0,2289	-0,0057	0,0669	0,0420	-0,1417	-0,0284	-0,2479
RDS	0,1166	<,0001	<,0001	<,0001	<,0001	<,0001	0,0004	<,0001	0,1339	1,0000	-0,8361	-0,6175	-0,2966	-0,4984	0,3625	-0,0245	-0,4052	0,3576	0,3851	-0,4393
SDS	0,0174	0,0877	0,0086	0,0334	0,0304	0,0522	0,0776	0,0383	0,8107	<,0001	1,0000	0,0849	0,1074	0,3972	-0,5482	-0,0233	0,4392	-0,2119	-0,5560	0,7025
RS	0,5813	<,0001	<,0001	<,0001	<,0001	<,0001	0,0003	<,0001	0,0006	0,0013	0,6933	1,0000	0,3848	0,3357	0,1343	0,0761	0,0945	-0,3312	0,1054	-0,2163
1047/1022	0,8901	0,0679	0,0447	0,0965	0,0415	0,1242	0,3357	0,1231	0,0197	0,1593	0,6176	0,0633	1,0000	-0,0960	0,1442	0,0711	-0,0372	-0,1156	0,1255	-0,2847
995/1022	0,5604	0,0499	0,1119	0,0185	0,2088	0,0088	0,0617	0,0157	0,2819	0,0132	0,0546	0,1087	0,5774	1,0000	-0,2921	-0,1967	0,4345	-0,2001	-0,2243	0,3407
α-helix	0,1748	0,7492	0,5521	0,8464	0,8068	0,6511	0,8658	0,9730	0,9793	0,0892	0,0068	0,5412	0,4086	0,0887	1,0000	0,2658	-0,4051	-0,3849	0,9280	-0,2834
β-sheet	0,2027	0,4151	0,5722	0,5712	0,2645	0,7106	0,1038	0,3127	0,7618	0,9116	0,9158	0,7301	0,6847	0,2575	0,1227	1,0000	-0,7500	-0,2504	-0,1120	-0,0812
Random coil	0,0086	0,0316	0,1039	0,0319	0,0251	0,0851	0,0042	0,0240	0,8490	0,0551	0,0360	0,6679	0,8318	0,0091	0,0158	<,0001	1,0000	-0,3109	-0,1314	0,2605
Turn	0,0856	0,0229	0,2492	0,0278	0,0574	0,0157	0,0518	0,0413	0,5190	0,0938	0,3317	0,1227	0,5084	0,2491	0,0224	0,1468	0,0691	1,0000	-0,2951	-0,0969
α/β ratio	0,3664	0,5339	0,6817	0,9998	0,5097	0,5613	0,4437	0,7019	0,8975	0,0696	0,0059	0,6321	0,4724	0,1952	<,0001	0,5217	0,4516	0,0853	1,0000	-0,2671
Amide I / Amide II	0,0078	0,5802	0,3780	0,4713	0,5867	0,3388	0,3786	0,2564	0,2540	0,0360	0,0002	0,3215	0,0974	0,0452	0,0990	0,6430	0,1307	0,5796	0.1208	1,0000

Above the main diagonal the correlations are shown, and below the main diagonal the p-values are displayed. WAC: water absorption capacity, OAC: Oil absorption capacity, WAI; Water absorption index, WSI: Water solubility index, SP: Swelling power, RDS: Rapid digestible starch, SDS: Slow digestible starch, RS: Resistant starch, IVPD: *in vitro* protein digestibility.
